# Periodic catatonia with long-term treatment: a case report

**DOI:** 10.1186/s12888-017-1497-6

**Published:** 2017-09-29

**Authors:** Ruei-An Chen, Tiao-Lai Huang

**Affiliations:** Department of Psychiatry, Chang Gung Memorial Hospital– Kaohsiung Medical Center, Chang Gung University College of Medicine, 123, Ta-Pei Rd, Niao-Sung, Kaohsiung, 833 Taiwan

**Keywords:** Catatonia, Lorazepam, Daylight exposure

## Abstract

**Background:**

Periodic catatonia has long been a challenging diagnosis and there are no absolute guidelines for treatment when precipitating factors are also unclear. We report a schizophrenia patient with periodic catatonia with a 15-year treatment course. A possible correlation between decreased daylight exposure and periodic attacks has been observed.

**Case presentation:**

We describe a 49-year-old woman with periodic catatonia associated with schizophrenia with 15 years of follow-up. The patient was treated with the antipsychotics risperidone, haloperidol, loxapine and quetiapine, but catatonia still relapsed once per year during the first few years of her disease course. The treatment was consequently been switched to clozapine due to fluctuated psychotic illness, and a longer duration of remittance was achieved. Lorazepam-diazepam protocol was used for rapid relief of catatonic symptoms, and was able to significantly shorten the duration of the symptoms. In addition, we observed a possible correlation between catatonic episodes and decreased daylight exposure during the 15-year duration.

**Conclusions:**

Successful treatment of acute periodic catatonia was achieved with a lorazepam-diazepam protocol, and the patient remained in remission for a longer duration under clozapine treatment. Besides, the possibility of decreased daylight exposure acting as a precipitating factor was observed during our 15 years of follow-up.

## Background

Catatonia is an extreme movement disorder with clinical syndromes characterized by stupor, immobility, mutism, mannerism, negativism and agitation. The syndrome was initially described by Kahlbaum in 1874 [1], now affects a great amount of patients with affective illness and schizophrenia, who accounts for 25% of the catatonia population [2]. An untreated and prolonged course of catatonic features may lead to life-threatening complications such as the development of pulmonary embolism [3] or aspiration pneumonia, and significantly increased mortality and morbidity.

Even though the pathogenesis of periodic catatonia is still poorly understood, early intervention and identification of immunological, biological or environmental risks factors are crucial once catatonic features are observed. It is reported that the average interval of recurrent catatonia is 10.7 months, varying from 4.5 to 20 months [4], but there are few data on the intervals of periodic catatonia or the correlation between catatonic episodes and changes in climate. Besides, current reports on long term follow up on patient’s condition are rare.

Benzodiazepams had long been considered the first line of treatment for catatonic symptoms [5, 6]. Huang et al. [7] had introduced the lorazepam-diazepam protocol since 2005. The protocol is utilized with one ampule of Lorazepam 2 mg/ml intramuscular injection once catatonia is detected. Follow by second Lorazepam 1 ampule dose after 2 h. If catatonic symptoms had not resolved, then intravenous infusion of 1 ampule of Diazepam 10 mg/ml diluted in 500 ml normal saline with infusion rate of 80 ~ 100 ml/h every 8 h should be administered until catatonia subsided. The technique had been widely used in Chang Gung Memorial Hospital since 2005 as a quick resolvent of catatonia.

In this report, we describe the case of a periodic catatonic patient with a follow-up period of 15 years under antipsychotic treatment with clozapine, whose catatonic episodes mostly occurred in the autumn and winter.

## Case presentation

### First episode

This 49-year-old single, unemployed woman with schizophrenia was diagnosed at the age of 33. She had a younger brother who was also diagnosed with schizophrenia at young age and had no children. She was able to served as an administrative clerk in government agency before her social and occupational began to declined as the result of schizophrenia which had led to long term occupational and economic problems. She had been admitted to the psychiatric hospital for twelve times due to fluctuated psychotic symptoms. However, hospitalization due to catatonia attack had accounts for seven out of the twelve admissions. One and a half year after the first episode of schizophrenia, the patient was hospitalized due to auditory hallucination with voice commanding which led to catatonic features manifesting with mutism, stupor, rigidity and eyelid negativism. Bush–Francis Catatonia Rating Scale (BFCRS) score [8] was 15. Details of clinical treatment courses are listed on Table 1. Schizophrenia and catatonia were diagnosed according to the DSM-V criteria.

**Table 1:** Clinical presentation of catatonic symptoms

Episode	Admission date	age	Catatonic symptoms	Positive symptoms	Medications	Lorazepam-diazepam protocol
1	2001/8/31	35	Stupor, negativism, mutism	AH	Risperidone 4 mg/day	Diazepam IVD 10 mg diluted in 500 ml Normal saline for 3 days
2	2002/12/16	36	Stupor, negativism, mutism	AH, Capras syndrome	Haloperidol 15 mg/day ➪ Olanzapine 15 mg/day Lorazepam 3 mg/day	Diazepam IVD for 2 days
3	2003/9/24	37	Stupor,catalepsy, mutism	AH	Loxapine 150 mg/day Lorazepam 2 mg/day	Lorazepam 2 mg IM follow by another dose of Lorazepam 2 h later
	2003/ 10/11		mutism, stupor	AH		Lorazepam 2 mg IM for three dosage with interval of 2 h
4	2004/2/6	38	Stupor, catalepsy, mutism	AH, Bizarre behavior, Capras syndrome	Quetiapine 550 mg/day Diazepam 2 mg/day	Not administered
5	2006/2/3	40	Stupor, mutism, catalepsy	VH, AH, delusion of being controlled, bizarre delusion	Clozapine 200 mg/day Lorazepam 2 mg/day	Lorazepam 2 mg IM for three dosage with interval of 2 h
6	2006/9/25	40	Stupor, negativism, mutism	AH, persecutory delusion, self harm behavior	Clozapine 200 mg/day Lorazepam 2 mg/day	Lorazepam 2 mg IM follow by another dose of Lorazepam 2 h later, follow by diazepam 10 mg diluted in N/S 500 ml for 3 days
7	2015/11/23	49	Stupor, mutism, agitation for 2 h, recurrent 6 h later	AH, persecutory delusion	Clozapine 300 mg/day Lorazepam 3 mg/day	Lorazepam 2 mg IM for three dosage with interval of 2 h

*Abbreviation*: *AH* auditory hallucination, *VH* visual hallucination, *IVD* Intravenous dripping, *IM* intramuscular, *N/S* normal saline

### Second episode

The second episode of catatonia occurred 1 year and 3 months after the first episode, with the clinical symptoms of mutism, negativism, staring to the front, hypoactivity and Capgras syndrome. BFCRS score was 18. Antipsychotic treatment was switched from haloperidol to olanzapine 15 mg/day due to poor medication response, and intravenous infusion of diazepam was administered in addition to oral-form lorazepam 3 mg/day. Slight verbal response with subsidence of negativism developed on the second day of admission. However, differing from the first one, this episode took place in the winter, with a daylight duration of 167.4 h per month in Kaohsiung, Taiwan, according to Central Weather Bureau records, which was the least amount of daylight for the year. The same pattern was observed in the following catatonic episodes.

### Third episode

The third episode of catatonia occurred the following year during late September.

Social withdrawal with unusual behavior, locking herself in the room all day long and poor verbal response gradually developed 9 months after she had been discharged from the hospital. Catalepsy and mutism were noticed after arriving at our emergency department with BFCRS score of 18, therefore the lorazepam–diazepam protocol with an initial injection of intramuscular lorazepam 2 mg was administered. Development of eye contact and some response by nodding the head occurred after the first dose of lorazepam. We administered the second dose of intramuscular lorazepam 2 mg 2 h later, and the mutism subsided 3 h after that. However, a sudden onset of rigidity of the four limbs, mutism, stupor and no eye contact was observed half a month after admission. The lorazepam–diazepam protocol was utilized again and the symptoms improved after one dose of intramuscular lorazepam 2 mg. The daylight duration was 192.6 h per month, which was also the least daylight exposure for the year, according to records.

### Fourth episode

Despite good antipsychotic medication adherence, the fourth episode of catatonia, manifesting with a decreased response to stimulus, mutism and staring to the front for whole day, had developed 4 months after the previous attack. Social withdrawal had been noted for 2 weeks prior to admission on January, 2004. BFCRS score was 14. Quetiapine 550 mg/day was administered upon admission, but the patient remained muted and refused to have meals for the next 13 days. The lorazepam-diazepam protocol was utilized this time due to a changeover to another attending psychiatrist. The catatonic symptoms remitted after 13 days. Again, the daylight duration was 183.2 h per month, the least for the year. A periodic pattern had been observed.

### Fifth and sixth episodes

Due to fluctuated psychotic symptoms, main regimen of schizophrenia was adjusted to clozapine 100 mg/day thereafter and the patient maintained good medication adherence. However, her occupational and social functioning had begun to deteriorate during recent catatonic attacks. Recurrent catatonic symptoms, including stupor, negativism, mutism and catalepsy, were noted 2 years after the administration of clozapine in late January 2006, with a daylight duration of 162.5 h per month, the second shortest duration for the year. BFCRS score was 15. She was thus readmitted. The mutism resolved after one dose of intramuscular lorazepam 2 mg. In addition, clozapine was increased to 200 mg/day to treat the prominent positive symptoms. Another attack of catatonia occurred 7 months later with manifestations of negativism, mutism, posturing and refusing to eat. She was admitted again and treated with the lorazepam–diazepam protocol. She started to develop a motor response after the initial injection of lorazepam 2 mg. We administered a second dose of lorazepam 2 h later and started intravenous dripping of diazepam 10 mg diluted in 500 ml normal saline. The posturing and negativism subsided on the second day, and a verbal response developed on the third day.

### Seventh episode

The patient remained symptom-free for the next 9 years under a clozapine 300 mg/day regimen, but was admitted again due to prominent positive symptoms in November 2015. However, progressive consciousness changed to stupor, mutism and severe agitation with self-harming behavior 3 months after the last discharge. Eyelid negativism had been observed upon her arrival at the hospital. BFCRS score was 16. The lorazepam–diazepam protocol was administered with intramuscular injection of 4 mg of lorazepam during the first 2 h. A few motor responses developed, and eyelid negativism subsided 2 h later, as the patient became more lucid after administration of lorazepam. However, the patient remained mute and withdrawal from food or drink. The catatonic state seemed to gradually resolve, but 5 h later, she returned to a stupor status with eyelid negativism and muscle rigidity. Intramuscular injection of lorazepam 2 mg was then administered. Intensity of rigidity had decreased after the third dose of lorazepam IM as we continued oral lorazepam 3 mg per day. Verbal response with vocabulary only developed on the second day of admission, along with improvement of muscle rigidity. She began eating on the third day and was able to engage with the interviewer, talking about the existence of auditory hallucination and persecutory delusion.

## Discussion

Periodic catatonia is an extremely rare type of catatonia, its diagnosis and management can be quite challenging. Ever since Huang et al. [6]reported the use of a lorazepam–diazepam protocol to relieve catatonic symptoms in schizophrenic patients, it has been proven to be an efficient treatment for acute catatonia. In our case, the lorazepam-diazepam protocol was used five times beginning in 2003 and the catatonic symptoms all resolved within 3 days. Furthermore, the catatonic attacks became less frequent after clozapine was administered in 2006. There are several reports on the benefit of clozapine for catatonic patients, showing an increase in the duration of the catatonia-free period [9].

The patient remained symptom-free for 9 years after the antipsychotic drug was adjusted from quetiapine to clozapine in 2006. Several studies have also indicated the efficacy of second-generation antipsychotics such as olanzapine [10, 11], risperidone [12], quetiapine [13] and clozapine [14].In our patient, the catatonic symptoms relapsed once a year due to a failure to respond to haloperidol, risperidone, quetiapine, olanzapine and loxapine, but were in remission for 9 years under clozapine.

There might be multiple predisposing factors for periodic catatonia associated with schizophrenia. Insufficient vitamin D level during winter has been observed in schizophrenic patients [15],and is associated with increased aging of cellular signaling and consequently an exacerbation of negative symptoms. A decrease in the level of brain-derived neurotrophic factor [16] and an increase in the level of serum C-reactive protein [17] also have been observed. Increased levels of pro-inflammatory factors such as C-reactive protein and interleukin-6 receptor, which are associated with psychiatric disease, have been observed during European winters [18]. However, there is still insufficient evidence showing the relationship between the level of brain-derived neurotrophic factor and periodic catatonic attacks.

In this case, we found a correlation between decreased daylight exposure and catatonia attacks, despite the patient having good medication adherence (Table 2). The second episode of catatonia occurred in December 2002. The daylight duration for that month was 167.4 h, which was the least for the year. The fourth and fifth episodes took place in January 2004 and January 2006, when the daylight duration was the least for each of the 2 years. A similar pattern was seen during the third episode in September 2003, when the daylight duration was the third least for the year, and the sixth episode occurred during September 2006, when the daylight duration was the second least for the year. However, we did not find a positive correlation between the episodes and the average temperature. The exact contributing factors for periodic catatonia and their correlation with decreased daylight exposure are still unknown; therefore, further studies are required.

**Table 2:** Daylight duration(hours) in Kaohsiung,Taiwan

	Jan.	Feb.	Mar.	Apr.	May	June	July	Aug.	Sep.	Oct.	Nov.	Dec.
2001	192.5	178.3	224.3	168.5	148.3	207.6	186.4	**233.4** ^**ab**^	140.2	206.7	179.7	166.3
2002	221.9	204.2	213.7	238.1	185.1	236.4	198.3	206.7	188.7	224.3	203.8	**167.4** ^**ab**^
2003	211.7	204.7	196.4	188.7	209.1	199.3	311	196.9	**192.6** ^**ab**^	221	167.2	216.4
2004	**183.2** ^**a**^	**193.9** ^**b**^	185.4	226.9	255.2	236.9	217.2	209.4	191.5	255.8	209.3	209.4
2006	**162.5** ^**a**^	**195.9** ^**b**^	192	178.6	206.5	191.4	184.3	224.6	**175.9** ^**ab**^	206.2	188.6	154.6
2015	199.8	194.8	204.6	259.7	213.6	328.7	218.7	175.0	208.7	193.2	**198.6** ^**ab**^	195.7

^a^month of catatonia onset

^b^month of admission

## Conclusion

In this case report, periodic catatonia associated with schizophrenia was successfully treated by following the lorazepam-diazepam protocol, and was maintained under a longer duration of remission with clozapine control. A possible correlation between a decrease in daylight exposure and the development of catatonic symptoms was observed, but the exact mechanism still needs to be identified.

## Abbreviations

AH: auditory hallucination
IM: intramuscular
IVD: intravenous dripping
N/S: normal saline
VH: visual hallucination
